# Focus group study on perceptions and information needs regarding vaccines targeting the older population: a cross-country comparison in four European countries

**DOI:** 10.1007/s11357-022-00682-5

**Published:** 2022-11-21

**Authors:** Manuela Dominique Wennekes, Renske Eilers, Antonella Caputo, Amandine Gagneux-Brunon, Riccardo Gavioli, Francesco Nicoli, Zoltán Vokó, Aura Timen, on behalf of the VITAL Consortium, Amandine Gagneux-Brunon, Anna Czwarno, Antonella Caputo, Atika Abelin, Aura Timen, Cristina Angelin-Duclos, Elisabeth Botelho-Nevers, Florence Baron-Papillon, Francesco Nicoli, Manuela Wennekes, Maria Syrochkina, Mart Stein, Paul Stephane, Renske Eilers, Riccardo Gavioli, Sibilia Quilici, Simon Lewin, Yan Sergerie, Zoltán Vokó, Debbie van Baarle, Jim Janimak

**Affiliations:** 1 National Coordination Centre for Communicable Disease Control, National Institute of Public Health and the Environment (RIVM), Bilthoven, the Netherlands; 2 Athena Institute, VU University Amsterdam, Amsterdam, the Netherlands; 3 Department of Chemical, Pharmaceutical and Agricultural Sciences, University of Ferrara, Ferrara, Italy; 4 Centre International de Recherche en Infectiologie, Team GIMAP, INSERM, U1111, CNRS, UMR530, Université de Lyon, Saint-Etienne, France; 5 Department of infectious Diseases, University Hospital of Saint-Etienne, Saint-Etienne, France; 6 Chaire PreVacCI, Université Jean Monnet, Saint-Etienne, France; 7 CIC-INSERM 1408 Vaccinologie, CHU de Saint-Etienne, Université Jean Monnet, Saint-Etienne, France; 8 Syreon Research Institute, Budapest, Hungary; 9 Center for Health Technology Assessment, Semmelweis University, Budapest, Hungary; 10 Department of Primary and Community Care, Radboud University Medical Center, Nijmegen, the Netherlands

**Keywords:** Vaccines, Infectious diseases, Older adults, Qualitative, Decision-making

## Abstract

The increasing life expectancy leads to more older adults suffering from infectious diseases. Vaccines are available against diverse infections such as influenza, pneumococcal disease, herpes zoster and tetanus. However, vaccine acceptance is crucial for optimal preventive effect. The objective of the study is to perform a cross-country analysis of the perceptions and decision-making behaviour of older adults regarding vaccinations and their information needs. Focus groups with older adults were conducted in four countries: France, Hungary, Italy and the Netherlands. Data were analysed using thematic analysis. Demographic characteristics of participants were gathered with a questionnaire. Influenza and tetanus vaccines were commonly known, as was the disease influenza. On the contrary, the awareness of the vaccines against pneumococcal disease and herpes zoster were low. Participants also expressed a need for more information on vaccines, such as possible side effects, contra-indications and duration of protection, emphasizing that information is a condition for decision-making on vaccination. General practitioners were found to be the most important in information provision on vaccines. Perceptions on vaccines, such as effectiveness, side effects and safety, as well as perceptions on infectious diseases, such as severity, susceptibility and experiencing an infectious disease, played a role in the decision-making of older adults on vaccines. More awareness of the information needs among older adults with regard to vaccines should be raised among general practitioners and other healthcare providers. This requires appropriate knowledge about the vaccines among healthcare providers as well as communication skills to meet the information needs of older adults.

## Introduction

Due to increasing life expectancy and decreasing fertility rate, the proportion of older adults, those aged 60 years and older [1], will continue to grow over the coming decades [2]. Older adults are more susceptible to infectious diseases because of co-morbidity and immunosenescence [3]. The current COVID-19 pandemic also particularly affects older adults. Over the first year of the pandemic (until November 8th, 2020), 88% of COVID-19-related deaths in Europe occurred in those aged 65 and over [4]. In response to the pandemic, several vaccines were developed, and it has been calculated that these vaccines prevented more than 450,000 deaths among older adults between December 2020 and November 2021 [5]. This pandemic thus highlights the significance of vaccination to prevent disease and mortality in specific risk groups, such as elderly.

However, COVID-19 is only one example of a vaccine preventable disease disproportionally affecting older adults. Other examples of infectious diseases that occur with a higher frequency in older adults are invasive pneumococcal disease [6], herpes zoster [7] and tetanus [8]. Moreover, annually 88% of deaths caused by respiratory problems resulting from influenza occur among older adults [9]. Vaccines are available against each of the infectious diseases mentioned, and vaccination programs are in place against influenza and pneumococcal disease in most European countries [10].

Vaccination has shown to be an effective measure in the prevention of infectious diseases and led to a significant decrease in both incidence [11–13] and mortality of vaccine preventable diseases [5, 14, 15]. Vaccinations also provide indirect protection by herd immunity which decreases transmission of the pathogen [16]. Prevention of infectious diseases by immunization of the older adult population decreases thus the risk of infection and with it the risk of complications. Vaccination could therefore contribute to healthy ageing [15]. Furthermore, preventing infectious diseases among older adults will reduce the burden on healthcare services.

To be successful in preventing diseases and hospitalizations, vaccines need to be accepted by the targeted population. However, vaccination rates of older adults remain low. For example, in 2017, only 44% of older adults aged 65 and over in the European Union received the influenza vaccine [17]. Moreover, it was shown that, aside from influenza vaccine, there is little knowledge of other vaccines among older adults [18]. Other reasons for sub-optimal vaccine uptake were lack of vaccine recommendations by healthcare professionals (HCPs), perceived risk of side effects, and incorrect beliefs about vaccines [19].

A previous study showed that older adults perceive vaccines to have a positive effect; however, there is a strong need for more information [18]. Factors such as disease severity and susceptibility were important for older adults in their decision-making process on vaccination [20].

There is little information on factors important in vaccination decision-making in a cross-country context. The aim of this study was therefore to identify the factors that influence the decision-making process of older adults on vaccines and their information needs, in a multi-country context, using focus-groups.

## Method

### Participants and procedures

Participants were recruited in four countries in Europe: France, Italy, Hungary and the Netherlands. In each country, four focus groups (FGs) were conducted between August 2019 and January 2020. One FG in each country was planned in a rural area, whereas other FGs took place in urban areas. The FGs in each country targeted specific age groups; 50–64, 65–69, 70+ and one mixed aged group from 50 years and over (FG in rural area). Each FG consisted of five to eight participants. The cut-off points of the age categories were chosen to gather more insight in the perceptions of older adults on vaccines for them in the following stages: prior to being eligible for vaccination, the early years of retirement and old age. An attempt was made to include older adults from various living settings, such as care homes and independent living.

Various strategies were used in each country to recruit participants for the FGs, such as contacting welfare organizations for older adults, distribution of flyers among pharmacies and primary care practices and advertisements on social networking websites such as Facebook®. Participants could be enrolled for the study by expressing their wish to participate to the researchers in the country of residence. The participation request was approved if they met the criteria of age and living area specified for the particular FG they enrolled for. That is, they lived in an urban area in case of an urban FG, whereas they came from a rural area when participating in the rural FG. Moreover, it was assumed that older adults enrolling for a specific FG met the age criterium for that particular FG. Subsequently, information letters and directions to the FG location were sent to the participants. FGs were held at different locations, such as the site of welfare organizations for older adults, community centres, general practitioner’s office, research institutes and universities. Informed consent was obtained prior to the start of the FG. Participants did not receive a financial reward for participating. Travel expenses could be reimbursed in all countries.

In each country the FGs were conducted by a native research team consisting of a moderator and an assistant to take notes. This made it possible to conduct all the focus groups in the native language, considering implicit cultural norms and values.

### Data collection

FGs were perceived as most appropriate for this research, because, compared to individual interviews, people’s views become clearer through their interaction with other participants [21]. The duration was approximately 2 h per FG. Prior to the FG, participants were asked to fill out a brief questionnaire including demographic characteristics such as age and occupation, adult vaccines received (including for travel and leisure) and the types and frequency of HCPs consulted with.

The focus group guide consisted of a semi-structured open-ended topic list and included the following diseases and their corresponding vaccines: influenza, pneumococcal disease, herpes zoster and tetanus. These vaccines are offered in vaccination programs or are candidate vaccines for programs in either one of the study countries. Furthermore, the focus group guide was based on the “Integrated change model” [22] that combines the determinants explaining decision-making. From this model, the categories were incorporated in the topic list: awareness, perceptions (of vaccines and infectious diseases), motivation, self-efficacy, social influences such as the role of HCPs in decision-making on vaccinations, intention to accept vaccination and information preferences. Furthermore, questions related to healthy ageing were asked: what it entailed for participants, what actions they undertake to achieve it and whether vaccination could be part of it.

### Data analysis

The data of the demographic questionnaires were entered in MS Excel, uploaded to IBM SPSS statistics (version 24.001), and analysed on cross-country level.

All FGs were recorded and transcribed verbatim in the native language, leaving out any information that could identify participants (e.g. names). The resulting transcripts were translated into English to perform a cross-country analysis. One researcher (MW) analysed all transcripts by applying thematic coding, whereas another researcher (RE) independently analysed 25% of the transcripts. Differences in coding were discussed until consensus was reached. The analysis was conducted using MAXQDA 2020 and consisted of open coding, axial coding and selective coding. In the stage of open coding, codes were assigned to all relevant passages. Subsequently, during axial coding, related codes were grouped together, forming the main themes. Finally, during selective coding, relations between themes were identified.

## Results

### Demographic characteristics of the participants

One hundred and thirteen participants (*n*=113) participated in 16 FGs. Table 1 contains the demographic characteristics of participants. Due to recruitment difficulties, each FG in France included participants of mixed aged starting from 50 years. In all countries, most participants were still employed, including volunteer work. The percentages of participants reporting health problems were relatively high in France (73%) and Hungary (64%), as compared to the Netherlands (35%) and Italy (28%). Nevertheless, only a small fraction of the participants in the four countries received informal care, varying from 16% in Italy to 0% in both Hungary and France (Table 1). Furthermore, most participants (*n*=105) visited the general practitioner (GP) at least once over the previous year. Medical specialists were also frequently visited, but less than the GP.

**Table 1:** Demographic characteristics of the participants

Country	Nr. of participants	Mean age in years (age range)	Male/female	Nr. (%) of participants currently working	Health status: nr. (%) of participants with health problems^a^	Nr. (%) of participants receiving informal care^b^
Italy	32	67 (50–84)	15/17	20 (63%)	9 (28%)	5 (16%)
The Netherlands	26	68 (57–81)	12/14	22 (85%)	9 (35%)	1 (4%)
Hungary	33	66 (51–87)	7/26	19 (58%)	21 (64%)	0 (0%)
France	22	68 (52–75)	14/8	12 (55%)	16 (73%)	0 (0%)
Total all countries	113	67 (50–87)	48/65	73 (65%)	55 (49%)	6 (5%)

^a^Health problems refer to the presence of physical and/or mental problems.

^b^Informal care refers to care that is provided by family/friends without a medical background.

**Fig. 1:**
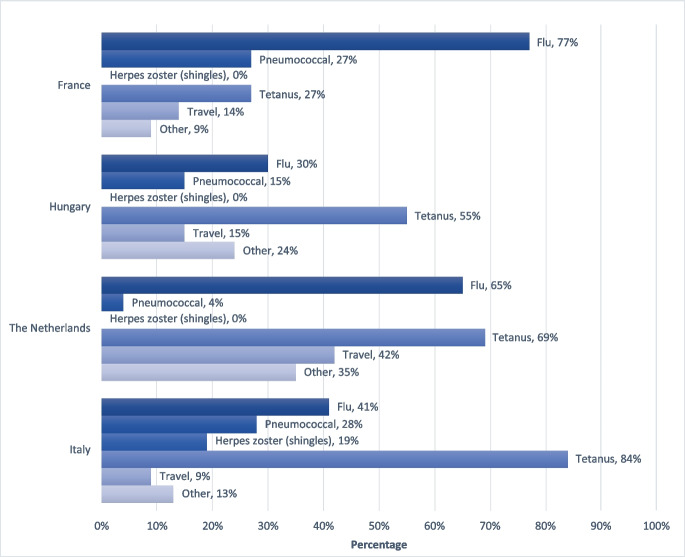
Vaccine uptake among the focus group participants per country

Most participants in the four study countries received influenza and tetanus vaccination over the course of their life, while pneumococcal and herpes zoster vaccine were much less common. Herpes zoster vaccination was only received by a small number of Italian participants. Travel vaccines were received by a minority of participants in each country. The category “other” refers to vaccines received earlier in life such as work related and military service (Fig. 1).

### Themes derived from the focus groups

**Fig. 2:**
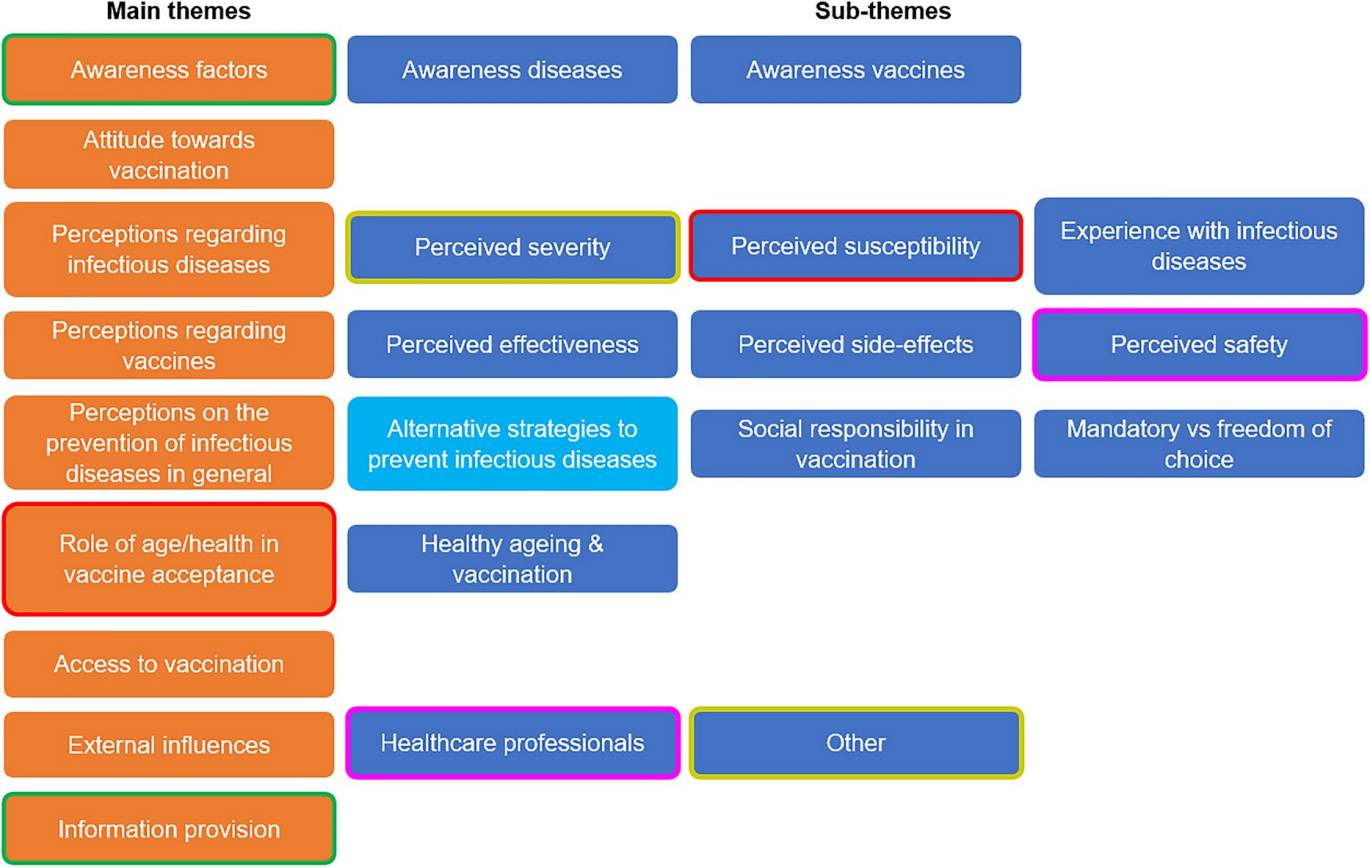
Cross-country analysis of the themes derived from the focus groups. The sub-themes found in all countries are dark blue, whereas pale blue indicates that the sub-theme ‘Alternative strategies to prevent infectious diseases’ was found in all countries, except Italy. The results of selective coding are indicated by blocks with a matching line around it. Thus, the themes “awareness” and “information provision” were related (green line), as were the sub-themes “perceived severity” and “other” (yellow line). In France only, the perceived safety and healthcare professionals were related (pink line)

An overview of the main themes and their sub-themes derived from the FGs is shown in Fig. 2. Main themes were awareness factors, attitude towards vaccination, perceptions regarding infectious diseases, perceptions regarding vaccines, perceptions on the prevention of infectious diseases in general, role of age/health in vaccine acceptance, access to vaccination, external influences and information provision. These are discussed using illustrative quotes from the FGs, emphasizing similarities and differences between the four countries of study.

From this point forward, statements that were not mentioned in all four countries are followed by the abbreviation(s) of the country/countries involved. Findings that are not followed by an abbreviation apply to all countries.

#### Awareness factors regarding the diseases and the vaccines

In general, the participants were aware of influenza and influenza vaccines. Similarly, the tetanus vaccine was also known by the participants, as were the circumstances requiring vaccination. However, this was less the case for the vaccines against pneumococcal disease and herpes zoster. Whereas a considerable number of participants experienced shingles, or knew someone who had, the herpes zoster vaccine was largely unknown in all countries. Moreover, whereas some of the participants in France and the Netherlands had heard little to nothing about pneumococcal disease, the vaccine against pneumococcal disease was much less commonly known by participants in all countries. Participants thus emphasized the need for more information on vaccines available (see “Information provision”).

#### Attitude towards vaccination

Most participants were positive about vaccines, describing them with qualifications such as “good” and “useful”. Reasons participants gave for accepting vaccines were based on the perceived possible benefits and absence of a perceived risk (Table 2, quotes 1–2). Furthermore, there were also participants mentioning that they intended to get a particular vaccine without providing any reasoning. Acceptance of the influenza vaccine was described as a habit (NL, FR). However, some participants had a negative attitude towards vaccines because they thought there were too many vaccinations (FR), did not believe in the influenza vaccine’s effectiveness (HU) or were suspicious towards new vaccines (IT). Furthermore, vaccination was described as a protection and prevention practice. The tetanus vaccine formed an exception regarding prevention because it could still be given after an injury (NL, FR, HU).

**Table 2:** Overview of illustrative quotes

Main theme	Sub-theme	Illustrative quote(s)
Attitude towards vaccination		1. *“Let them inject, if it doesn't help, it won't harm.”* (NL, 70+) 2. *“I do it too, why you never know.”* (IT, 50-64)
Perceptions regarding infectious diseases	Perceived severity of the disease	3. *“it [herpes zoster] is painful, it seems that someone is tearing you from the inside ...”* (IT, rural, mixed ages) 4. *“Because yes, I simply let myself be vaccinated. Because I think, I won’t die from the vaccine, but I might die from that flu* (NL, 65-69)
	Perceived susceptibility	5. *“Yes, then surely you would be more courageous to ask for it, if it showed that, yes, there is a good chance you would get it, you would go sooner.”* (HU, mixed ages) 6. *“I was not vaccinated because I said "I am not susceptible to flu"”* (IT, 70+) 7. *“ … there is always the idea that " this doesn't happen to me "…. There is always this thought.”* (IT, rural, mixed ages)
	Experience with infectious diseases	8. *“I am not against it, I mean if I get sick this year or the next, I will definitely get it the following year.”* (NL, rural, mixed ages)
Perceptions regarding vaccines	Perceived effectiveness to prevent disease and overall effects	9. *“I always refer to the professor, 50% success, no more. It's not huge but it's still worth the cost to try.”* (FR, mixed ages) 10. *“Nor does the seat belt provide 100 percent protection.”* (HU, 50-64)
	Perceived side effects	11. *“Well I think if you really induce a real disease with it, then I would say well then rather omit it. Because then I actually think what you offer, gives you more problems. Because that means, because if you, especially if you are in a vulnerable group, give it to people, that means that they will be made sick by you at that moment. Instead of giving something to prevent disease. And then I think yes, then you have to think three times before you try that.*” (NL, 65-69)
	Vaccine safety	12. *“You are taking a small, but real, personal risk by getting vaccinated. From a public health point of view, it is very effective, but for your personal case, it is possibly very bad.”* (FR, mixed ages). 13. *“it is not just being marketed uh nowadays, so it takes a lot of time and research and especially in the Netherlands, yes”* (NL, 50-64)
Perceptions on the prevention of infectious diseases in general		14. *“If it [disease] comes then it comes”* (NL, 70+) 15. *“of course you are allowed to die, but if you have a pneumonia, severe pneumonia and after that you are really much less and you would have had a choice in that, then I think you would have made that choice differently in advance.” (NL, 50-64)* 16. *“so, you actually have to be in advance with your own decisions like what would I want, what are the possibilities for that, is it also offered to me in it*” (NL, 50-64)
	Social responsibility in vaccination	17. *“And that I just don't know if I'd always want to do that. Because you can't protect the whole world and I don't want to protect the whole world.”* (NL, 50-64)
	Mandatory versus freedom of choice	18. *“when they are in nursing homes, it is an obligation. They have no choice.”* (FR, mixed ages)
Role of age/health in vaccine acceptance		19. *“my mother was 94 and at a certain point I think, yes you know, life is also finite at some point. And do you still have to have that woman vaccinated, for God knows what?* (NL, 50-64) 20. *“you have to die of something.”* (FR, mixed ages) 21. *“that are always pneumococci, that is an old man's friend. Because you can't die in a nicer, faster way than that way.”* (NL, 65-69) 22. *“I get the flu vaccine every year, as prescribed by the doctor because of various diseases ... I have enough of what I have and the flu or even a cold can be a problem for me.”* (IT, rural, mixed ages) 23. “*I never did it because for the moment I think my body can afford not to do it…”* (IT, 70+) 24. *“I do not do it now because I feel well”* (IT, 50-64) 25. *“She got the flu shot because she is at risk. Diabetic, etc. I have nothing, so I see no use.”* (FR, mixed ages) 26. *“but I wait until I will be 65 years old and then I will decide ... concerning prevention” (IT, rural, mixed ages)* 27. *“Yes, maybe that you then still and cherish your health more […] I hope not of course, but you will lose some of the physical health. And well, all in all, I think maybe you’ll be more open to indeed the call for such a flu vaccination”*. (NL, 65-69)
	Vaccination as part of Healthy Ageing	28. *“vaccinations are part of those important actions you can do to take care of yourself, and they allow you to arrive at old age certainly in a more discreet way” (IT, 70+)* 29. *“Healthy aging means more to yes ensuring that you keep moving, that you have good nutrition and things like that. Not with vaccinations.”* (NL, 70+)
Access to vaccination		30. *“for the flu vaccine, at one point the employers were proactive because it less disturbed the operation of the company.”* (FR, mixed ages)
External influences	HCPs	31. *“I think that compared to the vaccine, the medical world itself is not entirely in favour, so there might be dangers. They do not all agree, that means that there are risks. Or that the side effects are not entirely elucidated.”* (FR, mixed ages)
	Other	32. *“So, I think that anything that can help you feel more fit physically, not to run any risks, not to get into that pointer corner at work. That I would like to use that yes.”* (NL, 50-64) 33. *“but such a such a herpes, I have seen that with my own eyes when I worked in the health care among older adults, and I think yes you don’t wish that on anyone.”* (NL, 65-69) 34. *“He doesn't want to go get vaccinated because his mother, when she was alive, she had to die in '85, she got vaccinated and she got sick. So, he says, no!”* (FR, mixed ages) 35. *“I haven't met anyone who had serious side effects. What people see on Facebook and what they see in the press, I always like to highlight a press, if there is one, they are sure to push it and push it for a week so everyone knows.”* (HU, 50-64)
Information provision		36. *“Me, I tend not to bother my mind, I trust the doctor. They tell me for your well-being, it would be rather good for you to do that, it doesn't cost me much to do it.”* (FR, mixed age) 37. “*because when we have doctors or health care workers (HCW) who tell you ‘look, this is the path you must follow’ ... so convince yourself that to get to that famous old age we would all like to get to, this is the path you must follow. So it would be necessary also a little care from our side, to follow the doctors and to follow the HCW…*” (IT, 65-70)

#### Perceptions regarding infectious diseases

##### Perceived severity of the disease

Many participants agreed that the infectious diseases discussed were serious. A small number of participants perceived influenza as less severe for them or were unsure of its severity (NL, FR). Similarly, some participants doubted herpes zoster was a severe disease (FR). Some participants were also unaware if (HU) and how severe tetanus was (IT). Moreover, pneumonia/pneumococcal disease (IT, NL, HU), influenza (IT, FR) and tetanus (IT, FR) were perceived to be possibly fatal. Participants also mentioned implications such as loss of work (HU), and, in the case of herpes zoster, the risk that it will become a chronic condition (NL, FR). The perceived severity of herpes zoster was mainly due to the pain it causes (Table 2, quote 3).

For tetanus, the seriousness was not related to age (NL). Furthermore, participants said that tetanus was severe enough to vaccinate against (FR) and that this severity outweighed all possible side effects of the vaccine (HU).

The perceived severity of the disease motivated vaccine acceptance. This link was explained as a weighing of risks (IT, NL, HU) (Table 2, quote 4).

##### Perceived susceptibility

A part of older adults mentioned that they were not consciously thinking about their personal susceptibility (NL, FR). Participants did perceive older adults in general to be at increased risk for influenza (FR), pneumonia/pneumococcal disease (NL), herpes zoster (NL, FR) and tetanus (NL). Moreover, participants perceived that pneumonia often follows on an initial disease (HU), whereas experiencing influenza was also thought to increase susceptibility to other illnesses (NL). Also weakened health (NL) and social interactions (IT, HU) were perceived to increase susceptibility to infectious diseases.

Perceived susceptibility could also motivate vaccine-acceptance (Table 2, quote 5). Similarly, low perceived susceptibility could lead to decline vaccination (IT, HU) (Table 2, quote 6). Beliefs like “this will not happen to me” also played a role in perceived susceptibility (IT) (Table 2, quote 7).

##### Experience with infectious diseases

Experience with a certain infectious disease also played a role in vaccine acceptance; vaccination was often accepted after a disease experience (IT, NL, FR). Participants also indicated that they intended to accept the vaccine after they had first experienced the disease (IT, NL, HU) (Table 2, quote 8).

#### Perceptions regarding vaccines

##### Perceived effectiveness to prevent disease and overall effects

Vaccines were not only perceived as being effective in preventing infectious diseases, but also to have effect on organisms, as they take away the opportunity for the body “to clean itself” (NL) and there was fear that the weakened pathogen in the vaccine may not be weak enough (HU). Some positive effects were strengthening (FR) and training (HU) of the immune system and defence against the virus (NL, FR). Besides preventing the disease (IT, NL, FR), it was mentioned that vaccines could decrease the severity of the infectious disease, resulting in milder sick episodes (IT, NL, FR, HU) or a decreased risk of complications (HU).

Effectiveness of the influenza vaccine was perceived to be low. Participants mentioned they got influenza despite being vaccinated (IT, NL, HU). Inclusion of virus strains in the vaccine that did not match the influenza virus circulating was for some a reason to decline future influenza vaccinations (NL). Other participants, however, accepted that vaccines do not always work 100% (Table 2, quotes 9–10).

##### Perceived side effects

Participants indicated that side effects of a vaccine could lead them to reconsider acceptance of that vaccine in the future. Especially regarding vaccines for older adults, it was said that those causing illness were perceived to have too serious side effects to be accepted (NL) (Table 2, quote 11). However, participants do accept mild side effects (NL, FR, HU), such as headache. Some participants also said that they would accept a vaccine regardless of whether side effects were in proportion to the severity of the disease (NL).

##### Vaccine safety

Especially in France and to a lesser extent in Italy and Hungary, vaccines were perceived as potentially dangerous (Table 2, quote 12). Other participants perceived vaccines as safe because vaccines are researched (NL) and tested (IT) before they become available (Table 2, quote 13).

#### Perceptions on the prevention of infectious diseases in general

Perceptions that played a role were belief in vaccines, fate and anticipated regret. It was mentioned that it was important to believe in the vaccine’s effectiveness because faith in the vaccine was believed to increase the chance of remaining healthy (NL). Others had an opposing view instead, feeling that getting an infectious disease was inevitable (NL) (Table 2, quote 14).

Anticipated regret was also mentioned (NL, FR) (Table 2, quote 15). Another, closely related concept, concerned people who received the vaccine and did not contract the infectious disease. They felt unsure if their continued good health was due to the vaccine they received or not (NL, HU). It was mentioned that it was difficult to look back afterwards and that it was therefore important to make decisions on vaccination in advance (NL) (Table 2, quote 16).

##### Alternative strategies to prevent infectious diseases

Alternative strategies included additional measures to protect against infectious diseases, such as wearing a face mask (NL, FR, HU) and washing hands (HU). Moreover, it also included experiencing a disease episode to create antibodies against it (NL, FR, HU). Alternative medicine, such as homeopathy, was mentioned often among French participants. Part of them felt that alternative medicine could replace vaccines, whereas others felt that vaccination and alternative medicine should be combined.

##### Social responsibility in vaccination

Participants agreed that they would accept vaccination to protect others and/or to avoid infecting others. French participants felt that this social aspect of vaccination pertained especially HCPs as they could protect others by accepting vaccines. However, this social responsibility was also opposed (NL) (Table 2, quote 17).

##### Mandatory versus freedom of choice

There was disagreement between participants on whether vaccines should be mandatory or remain a free choice. Some felt that freedom of choice was too optional (NL), whereas others felt that a certain degree of freedom is important to a person (NL). Moreover, making vaccines mandatory was perceived as dictatorship (FR).

However, whereas older adults living independently are often free to make their own choice on vaccine acceptance, it was mentioned that this was not the case for those living in nursing homes (FR) (Table 2, quote 18).

#### Role of age/health in vaccine acceptance

Usefulness of vaccines in old age was also discussed (NL, FR). Some participants wondered whether there should be a certain age after which we no longer vaccinate people (NL, FR) (Table 2, quotes 19–20). However, other participants felt that there should be no upper age limit in offering vaccines (NL, FR) and that nobody has the right to decide that someone is too old to be offered a vaccine (NL).

In this discussion, current health status was also mentioned as a factor in determining the usefulness of vaccination in old age. When someone in old age was still healthy, vaccinating was perceived as useful. However, when one is already in ill health, death through an infectious disease, such as pneumococcal disease, might come as a relief (NL) (Table 2, quote 21). Vaccination should increase the quality of life, rather than only prolong it (NL). Some participants also mentioned that they accept the influenza vaccine because they have pre-existing medical problems (IT, NL, FR) (Table 2, quote 22). However, participants who feel that their body can overcome the disease (IT, FR) or are in good health did not perceive a need for vaccination (Table 2, quotes 23–25).

On the other side of the spectrum, a relatively young age was a reason to decline vaccination (Table 2, quote 26). Nevertheless, these participants indicated that they may think differently about vaccination in a couple years (Table 2, quote 27). Influenza (FR, HU), pneumococcal disease (NL, HU) and herpes zoster (FR) were also perceived to be more severe for older adults compared to younger individuals. Furthermore, participants felt it takes more time to recover from diseases as you get older (NL).

##### Vaccination as part of healthy ageing

Healthy ageing consists of mental, social and physical functioning (IT, FR, HU). Continuing to work was important (IT, FR), as were social relationships and sports. Participants were divided on whether vaccination could be part of healthy ageing. Some of them felt that vaccines could contribute to healthy ageing (Table 2, quote 28). Moreover, vaccines were perceived to have been beneficial, enabling people to reach old age (NL). Conversely, some participants felt that vaccines were not part of healthy ageing (NL, FR, HU) (Table 2, quote 29) or were unsure about whether vaccines could contribute to healthy ageing (FR).

#### Access to vaccination

Some participants wanted the influenza vaccine before they were eligible to receive it for free and therefore decided to pay for it (NL, FR). Moreover, it was argued that anyone who wants the influenza vaccine should be given it for free (NL). Costs of vaccines were also considered (IT, HU); for expensive vaccines, the acceptance depends on the perceived danger of the disease (HU).

Some participants mentioned that they were invited by their workplace for the influenza vaccination (NL, HU). Both participants (NL) and employers (FR) were positive about the possibility to receive it via their workplace (Table 2, quote 30).

Participants in all countries agreed that the current way of obtaining vaccines is easy. Only a part of the Italian participants mentioned bureaucracy as an obstacle to obtaining vaccines.

#### External influences

External influences contain parties such as HCPs, government/policies, family and experience of others with infectious diseases and/or vaccines.

##### HCPs

Participants experienced various behaviours from HCPs that encouraged vaccine-uptake: offering vaccine (HU), reminding patients (IT), checking whether patient was vaccinated (FR), convincing (IT), informing (IT, FR, HU) and recommending/advising patients (IT, FR, HU).

However, participants also mentioned that some HCPs showed a negative attitude and behaviour regarding vaccination, such as refusing to receive a vaccine themselves (FR), not reminding patients (FR), not advising/recommending vaccines (IT, FR) or even recommending/advising not to take a specific vaccine. Especially refusal by HCPs to receive a vaccine themselves creates suspicion and scepticism among participants regarding vaccines (FR) (Table 2, quote 31).

##### Other

Also, the social context plays a role in vaccine acceptance, such as the work context, family, media and others.

In the work environment, it was felt that illnesses should be avoided as much as possible to avoid critique from others, which led to willingness to accept the vaccine against influenza (NL) (Table 2, quote 32).

Regarding the influence from family members, it was mentioned that they could encourage vaccination (IT, HU), as well as discourage vaccination, by saying it is not necessary or by refusing to take them to the vaccination location (NL). Finally, it was mentioned that the decision to accept vaccination was independent of their spouse’s opinion (NL).

In addition, the experience of others with infectious diseases may encourage vaccine acceptance (Table 2, quote 33). Similarly, negative experiences from others with vaccines may prevent vaccine acceptance (FR) (Table 2, quote 34).

The media had multidirectional influences. A positive influence was that participants often heard of the existence of certain vaccines, such as herpes zoster, via the media (IT, FR, HU). Moreover, the arrival of the annual influenza vaccine is announced via the radio (HU) and much information on vaccines is received via the media (IT, FR, HU). However, the media also had negative effects, such as disturbance of attitudes on vaccines (FR), misinformation (HU), conflicting news on vaccines (IT) one-sided information (NL) and emphasize on severe but rare side effects (HU) (Table 2, quote 35).

#### Information provision

**Fig. 3:**
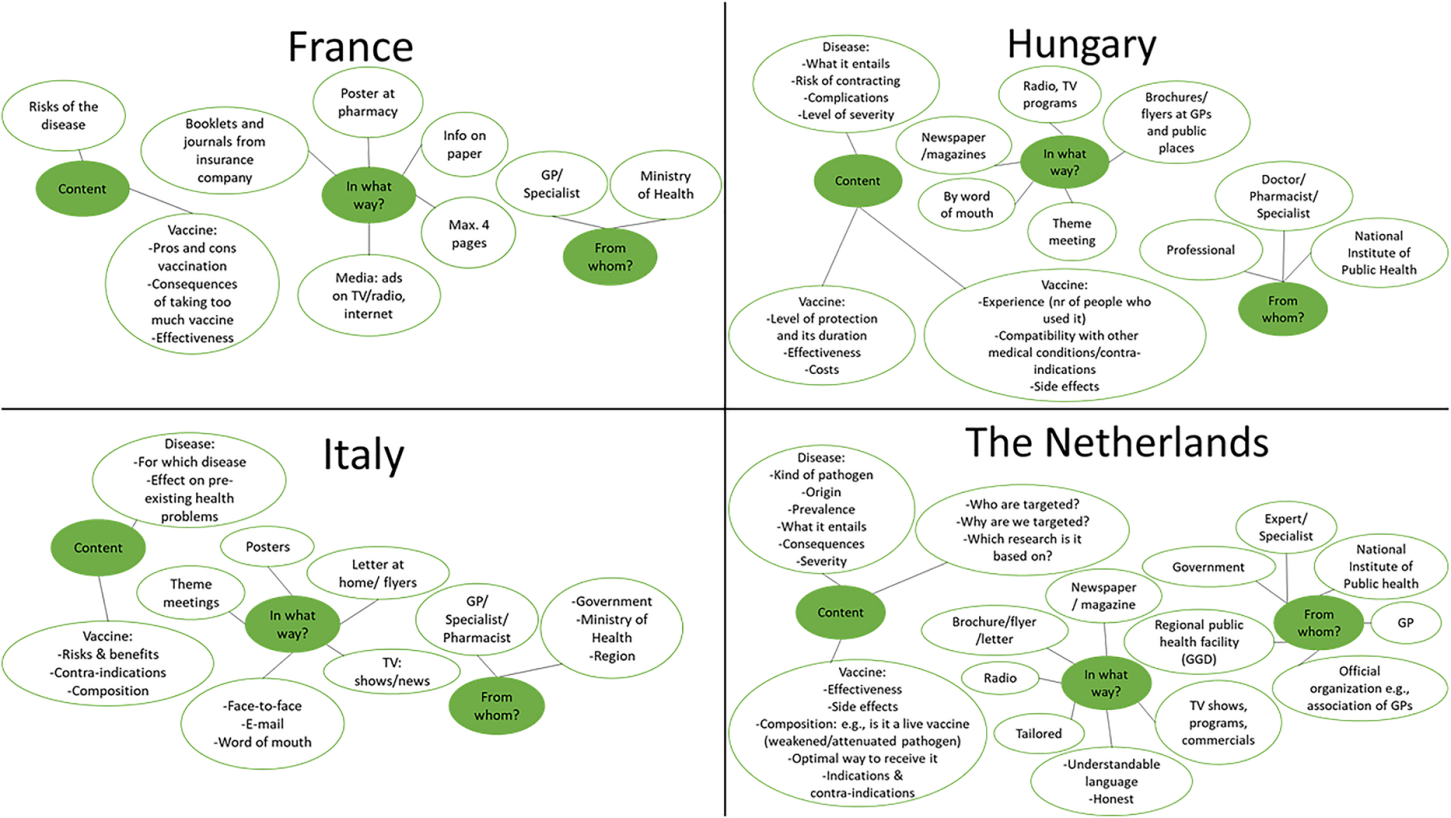
Examples of the information preferences of older adults regarding vaccinations. The green spots represent the three topics we asked participants with regard to their information preferences: (1) content of the message, (2) channel (what way) and (3) source (from whom). The white spots with a green line around it show participants’ information preferences regarding these three topics

Information provision was perceived to be very important and a condition for vaccine-acceptance. Figure 3 shows examples of the information preferences per country. Participants indicated their preferences regarding the source (from whom), channel (what way) and content of the message.

The preferred source of information was often the GP (IT, NL, FR), followed by other HCPs such as the medical specialist (IT, FR, HU), pharmacist (IT, HU) and doctor (HU). Participants felt that GPs should provide information on vaccines (IT, NL), propose relevant vaccines to patients (FR), remind patients to get boosters (FR) and recommend vaccines (IT). An important condition was that GPs had to be informed themselves on vaccines (NL). Moreover, in response to questions on the information provision and throughout the FG, participants frequently mentioned that they trust their HCPs regarding vaccines (IT, NL, FR). Furthermore, they also encouraged each other to trust HCPs on vaccines (IT, FR) (Table 2, quotes 36–37).

However, some participants experienced a negative patient-HCP relationship. Doctors were overburdened (HU) and had little time per patient (IT, FR). Furthermore, a lack of communication between patient and HCPs was mentioned (IT).

Preferred channels to receive information were the media, information letters at home (IT, NL) and flyers/brochures. The latter was disputed in Italy, where some thought this effective, whereas others thought the opposite. Furthermore, tailored information provision was a recurring theme (NL). This meant for example a general brochure with a link to a website with more in-depth information, also specifically for people with pre-existing medical conditions such as diabetes.

Regarding the content, participants wanted to receive information on the characteristics of the vaccine, such as its composition (IT, NL), side effects (IT, NL, HU), effectiveness (NL, FR, HU), duration of protection (HU) and contra-indications (IT, NL, HU). Moreover, participants also wanted information on the disease characteristics, such as what the disease entails (NL, IT, HU) and severity (NL, FR, HU). Finally, participants wanted also more general information on who was targeted and why (NL) and on the costs (HU).

## Discussion

Several main themes that affect vaccination decision making emerged from the data in the four countries including: awareness factors, attitude towards vaccination, perceptions (regarding infectious diseases and vaccines), role of age/health, access to vaccination, external influences and information provision. The cross-country analyses revealed differences within sub-themes such as questioning usefulness of vaccines in old age which was mentioned only in two (NL, FR) of the four countries. Two views emerged from this discussion: first, universal eligibility for vaccination regardless of age and, second, the perceived futility of prolonging lives of very old adults or older adults with serious morbidities without improving the quality of life. Furthermore, specifically French participants doubted the safety of vaccines while they frequently mentioned alternative medicine both as a substitute for vaccines and a form of treatment when ill.

A main finding was that influenza, the influenza vaccine and the tetanus vaccine, were commonly known by participants from all countries. However, the awareness of the vaccines against both herpes zoster and pneumococcal disease was low. Participants in all countries emphasized the need for more information on the vaccines available to them. This is also in line with findings of previous studies [18, 20]. Older adults can only choose to accept vaccines if they are aware of them. This finding becomes even more relevant considering that our study showed that the perceptions of both the infectious disease and the vaccine played a role in the decision-making of older adults on vaccines. For example, a high perceived severity of the infectious disease motivated vaccine-acceptance. To make an informed decision on vaccination, it is highly important that older adults are aware of the disease characteristics such as the severity. Moreover, experiencing an infectious disease, as well as the experience of someone else suffering from an infectious disease, was also a motivating factor to accept vaccination. For some, experiencing the disease seemed to be a pre-requisite to accept vaccinations.

Also, the perceived susceptibility, as well as participants’ current health, influences the vaccination decision of older adults. Furthermore, for some participants, experiencing side effects could lead to future decline of the vaccination. Age also played an important role; some older adults perceived themselves not yet sufficiently aged to need vaccines. Furthermore, participants within countries held different opinions on whether vaccination was part of healthy ageing. Future vaccination programs and campaigns should take this spectrum of opinions and considerations of older adults into account. The decision-making of older adults is multifactorial and complex. Not only does it depend on characteristics of the disease or characteristics of the vaccine, but it is also an interplay of age, current health and perceived susceptibility.

HCPs, and specifically GPs, were the preferred source for information on vaccines. Participants’ trust in the GP played an important role in this. Regarding the content of the information provision, participants wanted information on both the characteristics of the disease and of the vaccine. Participants preferred to be informed via media and flyers.

The current study had several strengths and limitations. Strengths of this study were that it explored the perspectives in four different socio-cultural contexts, enabling cross-country comparison. Furthermore, it was conducted by native research teams which made it possible to conduct the FGs in the native tongue of the participants. Also by using FGs, the views of participants were elicited and clarified through the interaction with the other participants, which enriches the data [21].

There were also some limitations. Due to recruitment difficulties, it was not possible in Hungary and France to conduct a FG in a rural area. In Hungary, there were no partners in the rural area who could provide the necessary facilities for the FGs and recruit participants. We could therefore only organize the FGs in the urban areas. Also in France, major difficulties were experienced in recruiting participants in the rural area. Therefore, in France and Hungary, all FGs were organized in the urban area. To mitigate this, we allowed older adults residing in rural areas to participate in the urban FGs. Moreover, in France, it was not possible to arrange the FGs for different age groups. Thus, all ages were represented in all FGs; however, this is not expected to have influenced the results. Moreover, the uptake of the influenza vaccine varied from 30% until 77% among the participants, whereas the mean uptake in Europe was 44% [17]. This means that participants who received the influenza vaccine were overrepresented in the Netherlands (65%) and France (77%). This may have resulted in selection bias due to overrepresentation of participants favourable towards vaccines. Moreover, in France, the number of participants suffering from comorbidities was very high, which might also explain the overrepresentation of individuals in the FG who received the influenza vaccination. This is in line with our findings indicating that pre-existing health problems promote vaccine acceptance among older adults. Finally, in the Hungarian FGs, women were overrepresented. This mirrored to some extent also the composition of the older adult population, where women are also in the majority. Other explanations for this overrepresentation might be that women are perhaps more interested in either health issues or research or had more spare time to participate.

The cross-country perspective used in this study showed that older adults’ decision-making on vaccines is more universal than expected, with participants in all countries emphasizing the need for more information on vaccines. With advancing age, the susceptibility to infectious diseases increases [6–8], as well as the mortality rate [9]. Lack of awareness among older adults regarding vaccines available thus takes away the opportunity to protect against these infectious diseases. Information provision by HCPs, and in particular GPs, on all vaccines available to older adults is therefore highly important. Moreover, we found that high perceived susceptibility promoted vaccine acceptance among participants, whereas low perceived susceptibility decreased vaccine acceptance. This is in line with previous studies [23, 24]. Therefore, in all countries, specific attention should be given to the role of age and current health, explaining the benefits of vaccination for the relatively healthy 60+ group. Particularly in France, attention should be paid to inform older adults on the safety of vaccines and educate on the effectiveness of vaccines over alternative medication. A specific effort should also be made to educate HCPs on the importance of vaccinating themselves as an example that vaccines can be trusted. More research is needed on the role HCPs perceive for themselves regarding providing information on available vaccines to older adults. It is necessary to verify whether HCPs are aware of the information needs of older adults on vaccinations.

When looking at the current COVID-19 pandemic, it has been found that people who relied on HCPs for their information on COVID-19 were more likely to refuse the COVID-19 vaccine as compared to those who received their information from mainstream news channels [25]. HCPs were often negative or unsure about receiving the COVID-19 vaccine themselves due to a perceived lack of data on the vaccine and potential risks that are not yet known [26]. Moreover, it has been shown that vaccine recommending behaviours of HCPs are associated with acceptance of vaccines for themselves, as well as their knowledge on vaccines [27]. Even though limited knowledge among HCPs on the relatively new vaccines against COVID-19 is understandable, previous studies also show limited knowledge among HCPs regarding other vaccines for older adults [28], as well as difficulty keeping abreast with vaccination recommendations [29]. Our findings show that information provision is critical in supporting informed decision-making of older adults regarding vaccines and that older adults prefer to receive information on vaccines from HCPs. It is therefore essential to educate HCPs thoroughly on the COVID-19 vaccine and the other vaccines available for older adults, such as against influenza pneumococcal disease, herpes zoster and tetanus. This should ensure that HCPs are well-equipped to inform their older adult patients and answer any questions they might have. The cross-country approach identified the aspects of both the infectious disease and vaccine that should be the focal point of generic information provision. The results of this study will therefore remain relevant in future pandemic outbreaks and introduction of new vaccines for older adults.

## Conclusion

Awareness of infectious diseases and the vaccines available to protect against these diseases is sub-optimal among older adults. This study identified the information needs of older adults regarding infectious diseases, as well as vaccines. These findings provide a starting point for interventions educating HCPs, and in particular GPs, on providing information about the available vaccines as well as the diseases they protect against. By meeting these information needs of older adults, HCPs enable them to make an informed decision on vaccine-acceptance.

## References

[1] World Health Organization (2020). Decade of healthy ageing: baseline report.

[2] Commission E (2020). Report on the impact of demographic change.

[3] Kline KA, Bowdish DME. Infection in an aging population. Curr Opin Microbiol. 2016. 10.1016/j.mib.2015.11.003. 10.1016/j.mib.2015.11.003 26673958

[4] World Health organization Regional Office for Europe. COVID-19 weekly surveillance report. Data for the week of 2-8 Nov (Epi week 45). 2020. Available at https://www.euro.who.int/__data/assets/pdf_file/0012/469677/Week-45-COVID-19-surveillance-report-eng.pdf Accessed 30 May 2022.

[5] Meslé MMI, Brown J, Mook P, Hagan J, Pastore R, Bundle N, Spiteri G, Ravasi G, Nicolay N, Andrews N, Dykhanovska T, Mossong J, Sadkowska-Todys M, Nikiforova R, Riccardo F, Meijerink H, Mazagatos C, Kyncl J, McMenamin J, Melillo T, Kaoustou S, Lévy-Bruhl D, Haarhuis F, Rich R, Kall M, Nitzan D, Smallwood C, Pebody RG. Estimated number of deaths directly averted in people 60 years and older as a result of COVID-19 vaccination in the WHO European Region, December 2020 to November 2021. Euro Surveill. 2021; 10.2807/1560-7917.ES.2021.26.47.2101021. 10.2807/1560-7917.ES.2021.26.47.2101021 PMC8619871 34823641

[6] European Centre for Disease Prevention and Control. Invasive pneumococcal disease. Stockholm: ECDC, 2020. Available at https://www.ecdc.europa.eu/sites/default/files/documents/AER_for_2018_IPD.pdf Accessed 7 June 2022.

[7] Pinchinat S, Cebrián-Cuenca AM, Bricout H, Johnson RW. Similar herpes zoster incidence across Europe: results from a systematic literature review. BMC Infect Dis. 2013. 10.1186/1471-2334-13-170. 10.1186/1471-2334-13-170 PMC3637114 23574765

[8] European Centre for Disease Prevention and Control. Tetanus. Stockholm: ECDC, 2020. Available at https://www.ecdc.europa.eu/sites/default/files/documents/Tetanus_AER_2018_Report.pdf Accessed 7 June 2022.

[9] Paget J, Iuliano AD, Taylor RJ, Simonsen L, Viboud C, Spreeuwenberg P. Estimates of mortality associated with seasonal influenza for the European Union from the GLaMOR project. Vaccine. 2022; https://doi-org.vu-nl.idm.oclc.org/10.1016/j.vaccine.2021.11.080. 10.1016/j.vaccine.2021.11.080 PMC8923032 35094868

[10] European Centre for Disease Prevention and Control. Vaccine scheduler: vaccine schedules in all countries in the EU/EEA. https://vaccine-schedule.ecdc.europa.eu/ Accessed 9 April 2022.

[11] Demicheli V, Jefferson T, Di Pietrantonj C, Ferroni E, Thorning S, Thomas RE, Rivetti A. Vaccines for preventing influenza in the elderly. Cochrane Database Syst Rev 2018;Issue 2. Art. No.: CD004876. 10.1002/14651858.CD004876.pub4. 10.1002/14651858.CD004876.pub4 PMC6491101 29388197

[12] Fedson DS, Nicolas-Spony L, Klemets P, Van Der Linden M, Marques A, Salleras L, Samson SI. Pneumococcal polysaccharide vaccination for adults: new perspectives for Europe. Expert Rev Vaccines. 2011. 10.1586/erv.11.99. 10.1586/erv.11.99 21810065

[13] Govaert TME, Thijs CTMCN, Masurel N, Sprenger MJW, Dinant GJ, Knottnerus JA (1994). The efficacy of influenza vaccination in elderly individuals: a randomized double-blind placebo-controlled trial. JAMA..

[14] Hak E, Buskens E, van Essen GA, De Bakker DH, Grobbee DE, Tacken MAJB, Van Hout BA, Verheij TJM (2005). Clinical effectiveness of influenza vaccination in persons younger than 65 years with high-risk medical conditions: the PRISMA study. Arch Intern Med.

[15] Esposito S, Franco E, Gavazzi G, de Miguel AG, Hardt R, Kassianos G, Bertrand I, Levant M-C, Soubeyrand B, Trigo JAL. The public health value of vaccination for seniors in Europe. Vaccine. 2018. 10.1016/j.vaccine.2018.03.053. 10.1016/j.vaccine.2018.03.053 29615269

[16] Randolph HE, Barreiro LB. Herd immunity: understanding COVID-19. Immunity. 2020. 10.1016/j.immuni.2020.04.012. 10.1016/j.immuni.2020.04.012 PMC7236739 32433946

[17] Eurostat. 44% of elderly people vaccinated against influenza https://ec.europa.eu/eurostat/web/products-eurostat-news/-/DDN-20191209-2 Accessed 6 January 2021.

[18] Albright K, Hurley LP, Lockhart S, Gurfinkel D, Beaty B, Dickinson LM, Libby A, Kempe A. Attitudes about adult vaccines and reminder/recall in a safety net population. Vaccine. 2017. 10.1016/j.vaccine.2017.11.001. 10.1016/j.vaccine.2017.11.001 29132991

[19] Johnson DR, Nichol KL, Lipczynski K. Barriers to adult immunization. Am J Med. 2008. 10.1016/j.amjmed.2008.05.005. 10.1016/j.amjmed.2008.05.005 18589065

[20] Eilers R, Krabbe PFM, de Melker HE. Motives of Dutch persons aged 50 years and older to accept vaccination: a qualitative study. BMC Public Health. 2015. 10.1186/s12889-015-1825-z. 10.1186/s12889-015-1825-z PMC4446004 25981624

[21] Kitzinger J (1995). Qualitative research. Introducing focus groups. BMJ..

[22] De Vries H, Mudde A, Leijs I, Charlton A, Vartiainen E, Buijs G, Clemente MP, Storm H, Navarro AG, Nebot M, Prins T, Kremers S. The European Smoking prevention Framework Approach (EFSA): an example of integral prevention. Health educ res. 2003;18(5):611-626. Available at https://www.jstor.org/stable/45100598 Accessed 7 June 2022. 10.1093/her/cyg031 14572020

[23] Eilers R, Krabbe PFM, de Melker HE. Factors affecting the uptake of vaccination by the elderly in Western society. Prev Med. 2014. 10.1016/j.ypmed.2014.10.017. 10.1016/j.ypmed.2014.10.017 25456809

[24] Kan T, Zhang J. Factors influencing seasonal influenza vaccination behaviour among elderly people: a systematic review. Public Health. 2018. 10.1016/j.puhe.2017.12.007. 10.1016/j.puhe.2017.12.007 PMC7111770 29408191

[25] Bhagianadh D, Arora K (2022). COVID-19 vaccine hesitancy among community-dwelling older adults: the role of information sources. J Appl Gerontol.

[26] Meyer MN, Gjorgjieva T, Rosica D (2021). Trends in health care worker intentions to receive a COVID-19 vaccine and reasons for hesitancy. JAMA Netw Open.

[27] Verger P, Fressard L, Collange F, Gautier A, Jestin C, Launay O, Raude J, Pulcini C, Peretti-Watel P. Vaccine hesitancy among general practitioners and its determinants during controversies: a national cross-sectional survey in France. EBioMedicine. 2015. 10.1016/j.ebiom.2015.06.018. 10.1016/j.ebiom.2015.06.018 PMC4563133 26425696

[28] Glenton C, Carlsen B, Lewin S, Wennekes MD, Winje BA, Eilers R. Healthcare workers’ perceptions and experiences of communicating with people over 50 years of age about vaccination: a qualitative evidence synthesis. Cochrane Database Syst Rev. 2021;7. 10.1002/14651858.CD013706.pub2. 10.1002/14651858.CD013706.pub2 PMC8407331 34282603

[29] MacDougall D, Halperin B, MacKinnon-Cameron D (2015). The challenge of vaccinating adults: attitudes and beliefs of the Canadian public and healthcare providers. BMJ Open.

